# Phosphoribosyl pyrophosphate synthetase activity affects growth and riboflavin production in *Ashbya gossypii*

**DOI:** 10.1186/1472-6750-8-67

**Published:** 2008-09-09

**Authors:** Alberto Jiménez, María A Santos, José L Revuelta

**Affiliations:** 1 Instituto de Microbiología Bioquímica and Departamento de Microbiología y Genética, CSIC/Universidad de Salamanca, Campus Miguel de Unamuno, 37007, Salamanca, Spain

## Abstract

**Background:**

Phosphoribosyl pyrophosphate (PRPP) is a central compound for cellular metabolism and may be considered as a link between carbon and nitrogen metabolism. PRPP is directly involved in the *de novo *and *salvage *biosynthesis of GTP, which is the immediate precursor of riboflavin. The industrial production of this vitamin using the fungus *Ashbya gossypii *is an important biotechnological process that is strongly influenced by substrate availability.

**Results:**

Here we describe the characterization and manipulation of two genes of *A. gossypii *encoding PRPP synthetase (*AGR371C *and *AGL080C*). We show that the *AGR371C *and *AGL080C *gene products participate in PRPP synthesis and exhibit inhibition by ADP. We also observed a major contribution of *AGL080C *to total PRPP synthetase activity, which was confirmed by an evident growth defect of the Δ*agl080c *strain. Moreover, we report the overexpression of wild-type and mutant deregulated isoforms of Agr371cp and Agl080cp that significantly enhanced the production of riboflavin in the engineered *A. gossypii *strains.

**Conclusion:**

It is shown that alterations in PRPP synthetase activity have pleiotropic effects on the fungal growth pattern and that an increase in PRPP synthetase enzymatic activity can be used to enhance riboflavin production in *A. gossypii*.

## Background

*Ashbya gossypii *is a filamentous hemiascomycete, which has been considered a paradigm of sustainable "white" biotechnology through its use in the industrial overproduction of riboflavin and other vitamins [1]. During the late growth phase, when maximum biomass has been reached, *A. gossypii *naturally exhibits high levels of riboflavin production as a detoxifying and protective mechanism [2]. However, we have recently described that riboflavin production can be enhanced considerably by genetic and metabolic engineering of the purine pathway, which provides the precursor for riboflavin biosynthesis [3,4].

**Figure 1:**
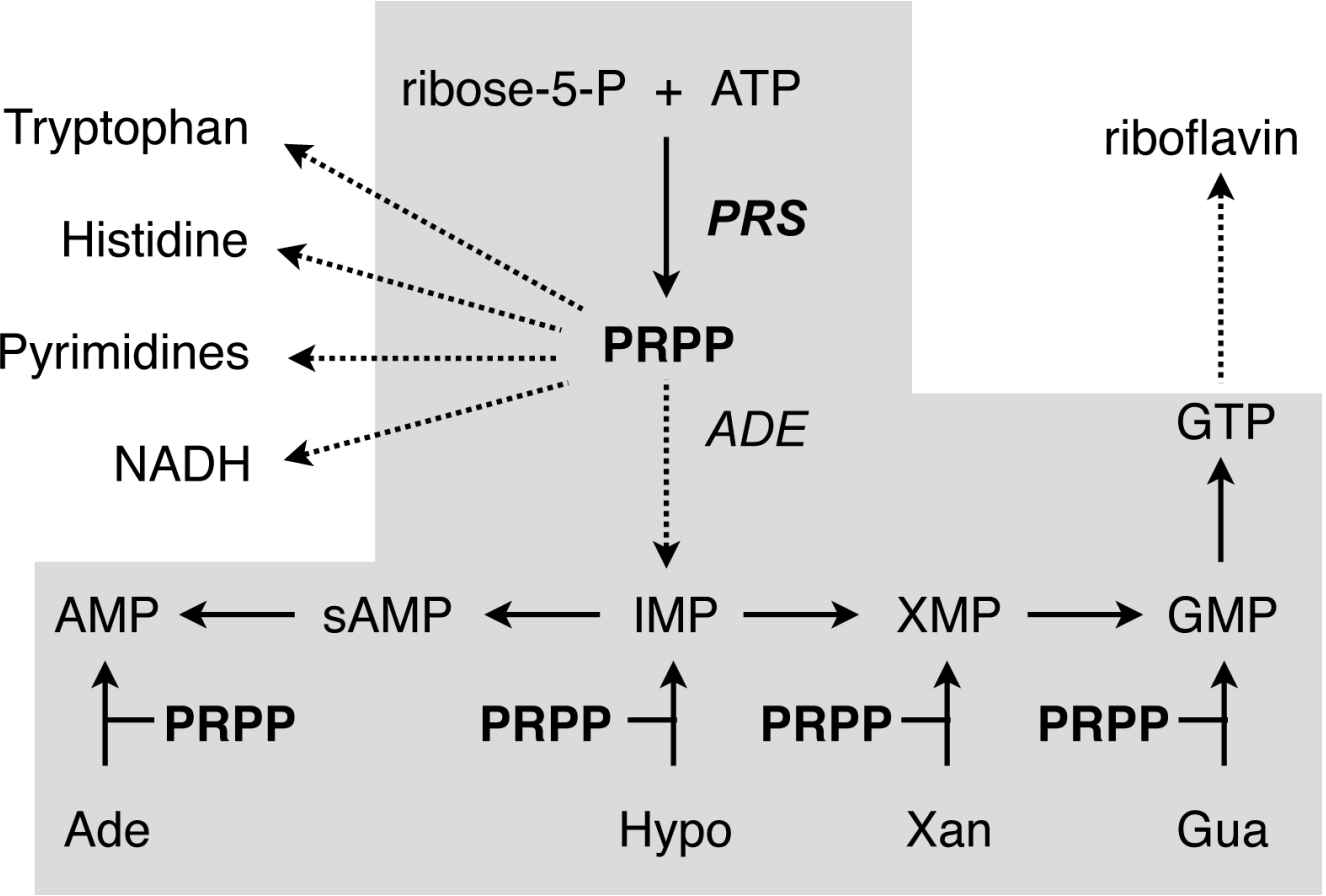
**Metabolic contribution of PRPP to the purine biosynthesis and other anabolic pathways**. The purine pathway is shaded; the *de novo *purine pathway starts with PRPP biosynthesis, while *salvage *pathways use PRPP to transform purine bases to monophosphate nucleotides. Dashed arrows indicate a multi-step pathway; sAMP, adenylosuccinate.

Riboflavin is synthesized from GTP and ribulose 5-phosphate through a multi-step pathway controlled by the *RIB *genes (*RIB1 *to *RIB5 *and *RIB7 *in *A. gossypii*) [5]. GTP, the immediate precursor for riboflavin production, is synthesized through the *de novo *purine pathway (Figure 1), which starts with the formation of PRPP. Alternatively, GTP can be formed through the purine *salvage *pathways (Figure 1), which recycle purines with the consumption of PRPP [6].

PRPP, synthesized from ribose-5-phosphate and ATP, is therefore a key compound for purine and riboflavin biosynthesis, and it is also an important cellular metabolite because it represents a link between carbon and nitrogen metabolism. PRPP is a biosynthetic precursor of histidine and tryptophan, and it is also required for the *de novo *and *salvage *pathways of purine, pyrimidine and pyridine (NAD^+^, NADP^+^) nucleotides. It has been calculated that approximately 80% of the metabolic flux through PRPP is directed to purine and pyrimidine synthesis [7].

The formation of PRPP is catalyzed by the enzyme PRPP synthetase, which is encoded by *PRS *genes. It has been shown that mutations in human *PRS *genes can either activate or inactivate the enzyme, leading to different hereditary disorders including hyperucemia, mental retardation, developmental delay, and other neurological pathologies [8-11].

According to the Ashbya Genome Database (AGD, ) [12] there are four putative annotated genes encoding PRPP synthetase in *A. gossypii*. In constrast, *Saccharomyeces cerevisiae *is equipped with a set of five unlinked *PRS *genes (*PRS1*-*PRS5*) [13,14]. Thus, in *S. cerevisiae *PRPP synthetase is organized in two interacting complexes or functional entities: a heterodimer comprising Prs1p-Prs3p; and a heterotrimer consisting of Prs2p-Prs4p-Prs5p [15]. Furthermore, systematic analyses of all possible combinations of *PRS *deletions in *S. cerevisiae *have revealed three possible phenotypes: synthetic lethality, when *PRS1 *or *PRS3 *deletions are combined with a disruption in *PRS5*, and the simultaneous deletion of *PRS2 *and *PRS4 *in either the *Δprs1 *or *Δprs3 *strains. A second phenotype is characterized by growth impairment and reduced enzymatic activity; this phenotype is found in mutants containing disruptions in *PRS1 *and *PRS3*, either together or in combination with *Δprs2 *or *Δprs4*. Finally a third phenotype, which only consists of a reduction in enzymatic activity, is encountered in single or combined deletions of *PRS2*, *PRS4 *and *PRS5*. This complex scenario can be explained in terms of the existence of three minimal subunits capable of sustaining the required PRPP intracellular pool in *S. cerevisiae*: namely, Prs1-Prs3; Prs2-Prs5 and Prs4-Prs5 [15].

Based on their enzymatic properties, so far three classes of PRPP synthetases have been described: Class I PRPP synthetases, which are dependent on phosphate ions for activity, are inhibited by purine ribonucleotide diphosphates and exclusively use either ATP or dATP as a diphosphoryl donors. PRPP synthetases from *Escherichia coli*, *S. cerevisiae *and mammals belong to class I [16-18]. Class II enzymes are specific from plants and are characterized by their independence of phosphate ions, their lack of allosteric inhibition, and their broad specificity for diphosphoryl donors [19]. Finally, a class III enzyme has recently been described in the archaeon *Methanocaldococcus jannaschii*, which is activated by phosphate and uses ATP as diphosphoryl donor, but lacks an allosteric site for ADP [20]. In a recent publication, a novel allosteric site for SO_4_^2-^, whose residues are strictly conserved in eukaryotic class I enzymes has been reported to stabilize the active site of PRPP synthetase [21].

Here we report the characterization of *AGR371C *and *AGL080C *genes encoding PRPP synthetases from *A. gossypii *and their effects on riboflavin production and growth. We found that PRPP synthetases from *A. gossypii *are inhibited by ADP. Furthermore, by combining *AgPRS *gene deletions we were able to define three different phenotypes that mimicked those previously encountered in yeast *Δprs *mutants. Finally, we describe the metabolic engineering of *A. gossypii *strains whose PRPP synthetase regulatory properties have been modified to significantly enhance the productivity of riboflavin.

## Methods

### Strains, media and techniques for A. gossypii culture

The *A. gossypii *ATCC 10895 strain was used and was considered as a wild-type strain. *A. gossypii *was cultured at 28°C using MA2 rich medium [22], synthetic complete media [23] or synthetic minimal media [24]. A concentration of 250 μg/ml of geneticin (G418) (Sigma, Steinheim, Germany), or 200 μg/ml of hygromycin B (Phytotechnology Laboratories, Shawnee Mission, USA), was used when specified. *A. gossypii *transformation, genomic DNA and RNA isolation, Southern-blot and northern-blot analyses, spores isolation, cell protein extraction, and HPLC determination of total riboflavin contents were carried out as previously described [24,25].

### PCR-based cloning of AGR371C and AGL080C genes from A. gossypii

Based on the annotated sequences for *AGR371C *and *AGL080C *in the AGD database [12], two pairs of primers were designed to PCR-amplify two genomic regions (approx. 3 Kb each) containing *AGR371C- *and *AGL080C- *coding DNA sequences. Both genomic fragments were cloned into a pBluescript-SK^+ ^vector (Stratagene) as *Kpn*I-*Hind*III (*AGR371C*) and *Eco*RI (*AGL080C*) fragments respectively. The resulting clones were shown to be correct by DNA sequencing of the entire fragments (data not shown).

### AGR371C and AGL080C gene disruption and overexpression

For *AGR371C *and *AGL080C *disruption, an integration cassette was constructed for each ORF (see below for further details). Briefly, for *AGR371C *disruption we obtained a *kanMX4 *selection module for geneticin resistance (*G418*^*r*^) with *Sal*I ends from the plasmid pAG-110 [26], which were subsequently treated with Klenow enzyme (Roche) to generate blunt ends. The *kanMX4 *blunt-ended fragment was inserted between *Hinc*II and *Eco*RV sites in the *AGR371C *ORF. The complete replacement module was obtained by digestion with *Nco*I and *Kpn*I and was used to transform spores of the *A. gossypii *ATCC 10895 strain. For *AGL080C *disruption, a hygromycin resistance (*Hyg*^*r*^) marker was obtained with *Bam*HI-*Kpn*I ends, which were subsequently treated with Klenow enzyme (Roche). The resulting *Hyg*^*r *^marker was inserted between two *Eco*RV sites present in the *AGL080C *ORF. Finally, the *AGL080C *disruption module was obtained by *Eco*RI digestion and was used to transform either spores of the *A. gossypii *ATCC 10895 strain, for the single disruption, or spores of the *A. gossypii *Δ*agr371c *strain, for the double disruption. Correct integrations were verified by analytical PCR and Southern-blotting experiments.

For the overexpression of different alleles of *AGR371C *and *AGL080C*, each ORF was inserted as an *Nde*I-*Bam*HI fragment into the overexpression cassette described below. Briefly, the overexpression cassette comprised: (i) a selection module for geneticin resistance, (ii) an integration module for stable integration of the cassette into the *AgLEU2 *locus, and finally (iii) an overexpression module based on the *AgGPD *promoter and terminator sequences, which have been reported to provide constitutive and high expression levels [3]. The overexpression modules were used to transform spores of the *A. gossypii *ATCC 10895 strain and positive clones were selected in media containing geneticin. Additionally, positive clones were verified for their leucine auxotrophy and analyzed by Southern-blotting.

### PRPP synthetase activity assay

A method previously described by Jensen *et al*. [27] was used for the quantification of PRPP synthetase activity. We measured the conversion of ^32^P-labelled ATP into ^32^P-labelled PRPP according to the following reaction:

^32^P-ATP + ribose 5-P → ^32^P-PRPP + AMP

The reaction mix contained 50 mM potassium phosphate, pH 7.5, 1 mM [γ-^32^P] ATP (10 Ci/mmol), 50 mM triethanolamine, 5 mM ribose 5-P, 5 mM MgCl_2_, 20 mM NaF, 15 mM phosphoenolpyruvate and 1 μmol/min of pyruvate kinase. We used 5–10 mg of total protein extract and the reaction was incubated at 28°C, taking 10 μl aliquots every 5 min over 1 hour. Samples were mixed with 5 μl of 0.33 M formic acid and immediately applied to a polyethylenimine-cellulose-coated plate (Sigma-Aldrich) for thin-layer chromatography. Separation of radiolabeled ATP and PRPP was carried out in 0.85 M potassium phosphate, pH 3.4. The radiolabeled spots corresponding to ATP and PRPP were cut and the associated radioactivity was quantified by liquid scintillation counting methods.

### Site-directed mutagenesis of AGR371C and AGL080C and inhibition of PRPP synthetase

Residue substitutions in the *AGR371C *and *AGL080C *ORFs were introduced by site-directed mutagenesis through PCR techniques using the primers listed in the Additional file 1. The inhibition assays were performed with different concentrations of ADP in the PRPP synthetase activity analyses. The inhibition rate was calculated as the percentage of specific activity in the presence of ADP with reference to the absence of ADP.

## Results

### Cloning and sequence analysis of the AGR371C and AGL080C genes from A. gossypii

We have previously shown that substrate availability is a limiting factor for riboflavin overproduction in *A. gossypii *and that increases in metabolic flux through the purine pathway significantly enhance riboflavin production [3,4,28]. PRPP is an important metabolite for purine biosynthesis because it is required in both the *de novo *and *salvage *pathways. This prompted us to wonder whether alterations in the PRPP intracellular pool might affect riboflavin production in *A. gossypii*.

**Figure 2:**
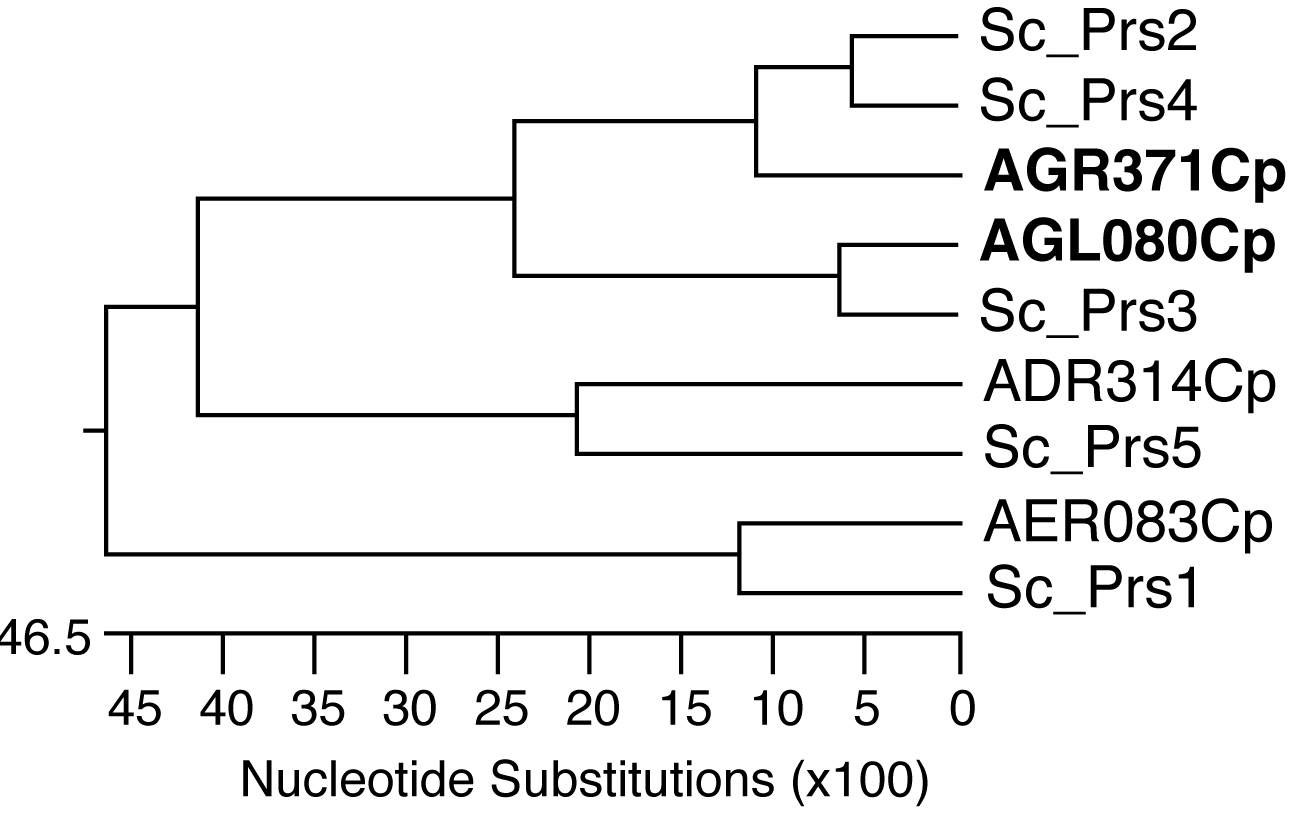
**Relationship between the *PRS *genes from *A. gossypii *and *S. cerevisiae***. Phylogenetic tree of the PRS proteins from *A. gossypii *and *S. cerevisiae*. The alignment of the protein sequences was performed using the W-CLUSTAL program included in the DNAStar package. In bold *AGR371C (AgPRS2,4) *and *AGL080C (AgPRS3)*.

In the AGD database there are four annotated genes encoding PRPP synthetase in *A. gossypii *that are syntenic homologues of the *PRS *genes from *S. cerevisiae*. A protein sequence alignment of *A. gossypii *and *S. cerevisiae *PRPP synthetases revealed a high degree of similarity (see Additional file 2). Furthermore, *AGR371C *is an ortholog of *ScPRS2 *and *ScPRS4*, suggesting that *AGR371C *is a common ancestor of both *ScPRS2 *and *ScPRS4*, which probably originated in a gene duplication event (Fig. 2). Thus, in accordance with the PRPP synthetase interacting complexes described in *S. cerevisiae *[15], we assumed that two homologous heterodimers might exist in *A. gossypii*, formed by Aer083cp-Agl080cp and Agr371cp-Adr314cp.

In order to study the specific contribution of each heterodimer to the overall PRPP synthetase activity and also to the production of riboflavin, for our next experiments we chose one member of each functional entity (*AGR371C *and *AGL080C*). For simplicity, *AGR371C *and *AGL080C *are henceforth referred to as *PRS2,4 *and *PRS3*, respectively, due to their homology with the *S. cerevisiae PRS *genes.

We amplified by PCR and cloned two genomic regions where *PRS2,4 *and *PRS3 *map and sequenced them. In good agreement with the nucleotide sequence deposited in the AGD database [12], *PRS2,4 *ORF comprised 957 bp encoding a protein of 318 amino acids, and *PRS3 *ORF consisted of 963 bp and coded for a protein of 320 amino acids. Both the Prs2,4 and Prs3 proteins contained most of the residues identified as being essential for catalysis, the maintenance of three-dimensional structure or protein-protein interactions in previously characterized PRPP synthetases [21,29] (see Additional file 2 for details).

Analysis of the 5'-non coding regions of *AgPRS2,4 *and *AgPRS3 *using the MatInspector program included in the GenomatixSuite 3.1.1 software  unveiled several TATA boxes (not shown) and, more importantly, one putative binding site for the transcription activator Bas1p in both the *AgPRS2,4 *and *AgPRS3 *sequences (positions -38 and -197 from ATG, respectively) (not shown). However, our previous work demonstrated that the expression of *AgPRS2,4 *and *AgPRS3 *is unaffected upon *AgBAS1 *deletion, suggesting that the presence of only one binding site is not sufficient to ensure transcriptional regulation by Bas1p [4].

### AgPRS2,4 and AgPRS3 gene disruptions affects PRPP synthetase activity and growth

**Figure 3:**
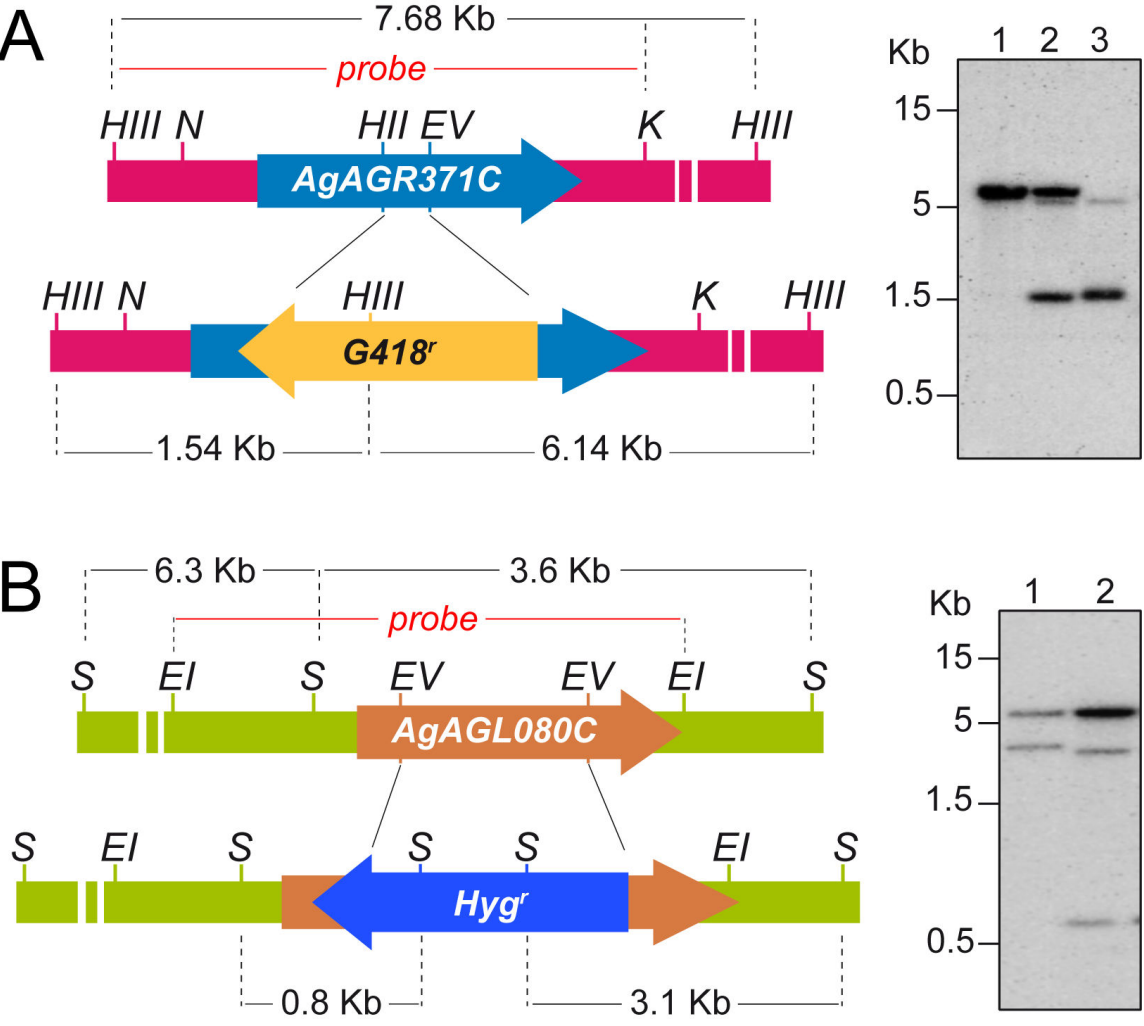
**Gene disruption of *AgPRS2,4 *and *AgPRS3***. A) Left, schematic representation of the strategy followed to achieve the disruption of the *AgPRS2,4 *gene (*AGR371C*). Right, Southern blot analysis to confirm correct *PRS2,4 *disruption. Genomic DNA was digested with *Hind*III and a genomic *Kpn*I-*Hind*III fragment was used as a radioactive probe: lane 1, wild-type strain; lane 2, heterokaryotic disruptant; lane 3, mutant Δ*prs2,4*. B) Left, schematic representation of the strategy for *AgPRS3 *(*AGL080C*) gene disruption. Right, Southern blot analysis to verify correct *PRS3 *disruption. Genomic DNA was digested with *Sac*I and a genomic *Eco*RI fragment was used as a radioactive probe: lane 1, wild-type strain; lane 2, mutant Δ*prs3*. HIII, *Hind*III; N, *Nco*I; HII, *Hinc*II; EV, *Eco*RV; K, *Kpn*I; EI, *Eco*RI; S, *Sac*I.

In order to elucidate the precise contribution of *AgPRS2,4 *and *AgPRS3 *to the overall PRPP synthetase activity in *A. gossypii*, we next disrupted both genes by inserting a dominant marker into each ORF. We used a *kanMX4 *selection module for geneticin resistance (*G418*^*r*^) to disrupt *AgPRS2,4 *and an hygromycin resistance (*Hyg*^*r*^) marker to disrupt *AgPRS3 *(Fig. 3). The final disruption modules comprised the *G418*^*r *^or *Hyg*^*r *^selection cassettes flanked by the *AgPRS2,4 *or *AgPRS3 *sequences, respectively, to enable homologous recombination and genomic insertion [30] (Fig. 3). With the disruption modules *A. gossypii *ATCC 10895 spores were transformed by electroporation as previously described [25]. Homokaryotic *G418*^*r *^and *Hyg*^*r *^transformants were obtained after sporulation and clonal selection of the primary heterokaryotic transformants. *AgPRS2,4 *and *AgPRS3 *disruption was confirmed by Southern-blot analysis using specific radiolabeled probes (Fig. 3).

**Figure 4:**
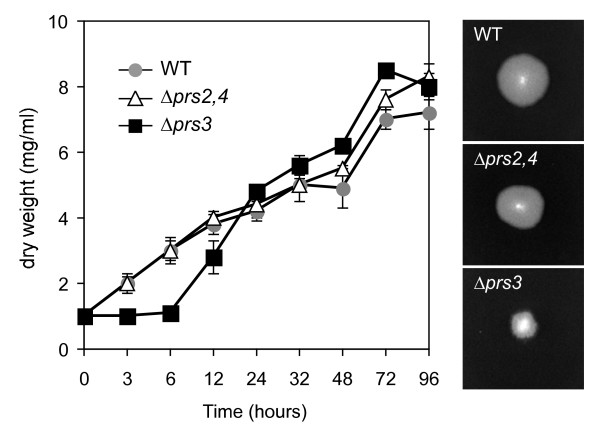
**Growth pattern of Δ*prs2,4 *and Δ*prs3 *mutant strains**. Left, *A. gossypii *wild-type and *prs *mutant strains were grown in liquid MA2 rich medium. At the indicated time-points, mycelia were harvested and weighed. Data are represented as an average of mycelium dry-weight per volume of culture. Error bars represent SD. Right, colony photographs of the wild-type, Δ*prs2,4 *and Δ*prs3 *strains grown on solid MA2 rich medium during 48 hours.

None of the *Δprs *mutants showed any nutritional requirement, confirming that the intracellular PRPP pool in both mutants was sufficient to support growth. Nevertheless, while the mutant *agΔprs2,4 *did not show any visible phenotype when grown on solid media, the *agΔprs3 *strain revealed a clear growth alteration, exhibiting mycelium-condensed and smaller colonies (Fig. 4). In addition, *agΔprs3 *displayed a significant reduction in the sporulation ability, this being 10-fold lower than that of the wild-type strain. We therefore analyzed liquid cultures of both *agΔprs2,4 *and *agΔprs3 *and determined the mycelial mass produced along growth. After four days of culture, the *agΔprs3 *strain displayed an initial growth delay with respect to the wild-type, although after 24 hours of culture its biomass was higher than that of the wild-type strain (Fig. 4).

**Figure 5:**
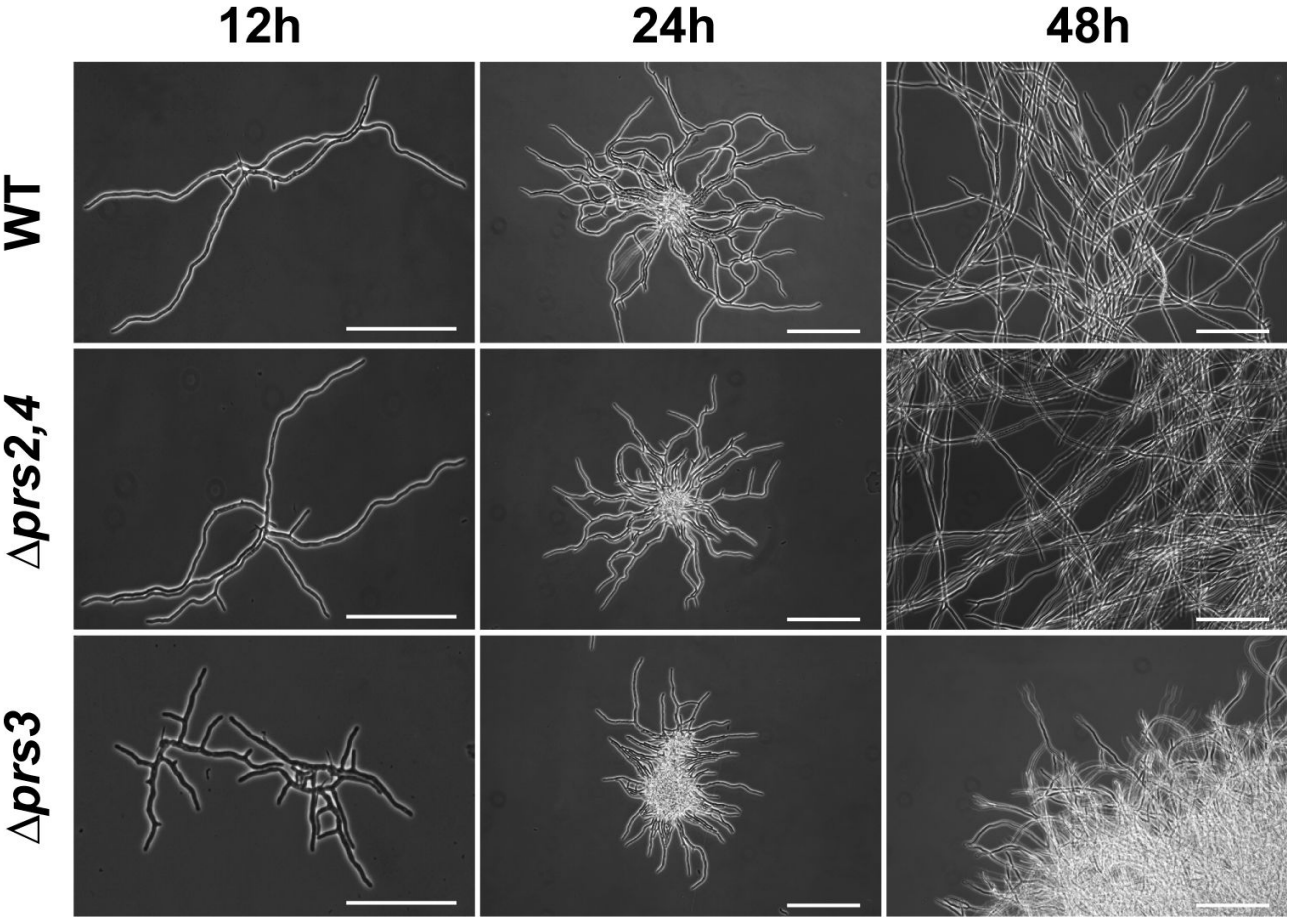
**Microscopic phenotype of *A. gossypii *wild-type, Δ*prs2,4 *and Δ*prs3 *strains**. Micelia of the *A. gossypii *wild-type, Δ*prs2,4 *and Δ*prs3 *strains grown on liquid rich medium were visualized under optical microscopy at 12, 24 and 48 hours of culture. Bar indicates 1 mm.

Next we wished to examine the growth alteration of *agΔprs3 *strain in greater detail by microscopic analysis. Again, *agΔprs2,4 *and wild-type strains were indistinguishable from each other, both of them showing an initial monopolar hyphal growth and a later bipolar growth, with branched hyphae (Fig. 5). Conversely, *Δprs3 *disruption caused a shortening on the hyphal length, and an abundant early branching was observed (Fig. 5). This higher branching index could explain the condensed mycelia of the *agΔprs3 *strain observed on solid media.

PRPP synthetase activity was altered in both *agΔprs2,4 *and *agΔprs3 *strains, but disruption of *PRS3 *resulted in a more marked decrease in the overall enzymatic activity, which in the *agΔprs3 *strain represented only 14% of the PRPP synthetase activity observed in the wild-type strain. Furthermore, riboflavin production was also partially impaired in the *agΔprs3 *mutant (Table 1). Taken together, these results suggest a major contribution of the *PRS3 *paralog to PRPP synthetase activity.

**Table 1:** PRPP synthetase activity and riboflavin production in the *A. gossyppi *strains used

**Strain**	**PRPP synthetase activity** **(nmol PRPP mg^-1 ^min^-1^)**	**Riboflavin** **(mg/L)**
Wild type (ATCC 10895)	10.0	28.0
*Δprs2,4 (agr371c)*	7.8	27.0
*Δprs3 (agl080c)*	1.4	20.7
*GPD-PRS2,4 (AGR371C)*	11.2	42.4
*GPD-PRS3 (AGL080C)*	17.0	40.4
*prs2,4-IQ*	12.2	48.6
*prs3-IQ*	17.9	51.6

In order to obtain a *Δprs2,4*-*Δprs3 *double mutant, spores of the *Δprs2,4 *strain were transformed with the *Δprs3 *disruption cassette described above. Selection of primary heterokaryotic clones was made in rich medium containing both geneticin and hygromycin. However, after clonal analysis of 200 homokaryotic strains derived from the previous heterokaryotic clones we failed to find any mutant strain showing resistance to both geneticin and hygromycin, suggesting that the double disruption of *PRS2,4 *and *PRS3 *may result in a synthetic lethal phenotype. To verify this hypothesis, we analyzed the initial heterokaryotic transformants (G418^r ^and Hyg^r^) by Southern-blotting and confirmed the presence of both single disruptant *Δprs2,4 *nuclei and double mutant *Δprs2,4*-*Δprs3 *nuclei (not shown). However, none of the homokaryotic transformants analyzed showed the double *Δprs2,4*-*Δprs3 *disruption. Additionally, the spores of the heterokaryotic *Δprs2,4*-*Δprs3*/*Δprs2,4 *were isolated by micromanipulation and analyzed individually; the same results described above were obtained. Apparently, the PRPP synthetase activity provided by *Δprs2,4 *nuclei is sufficient to maintain an adequate pool of PRPP and to allow the survival of the heterokaryotic transformants.

### AgPRS2,4 and AgPRS3 overexpression enhances riboflavin production

In a recent paper we reported that *PRS2,4 *and *PRS3 *mRNA levels did not change during the trophic and productive phases of *A. gossypii *growth. Furthermore, *PRS2,4 *and *PRS3 *show constitutive low expression levels, suggesting a housekeeping function of these genes [4].

**Figure 6:**
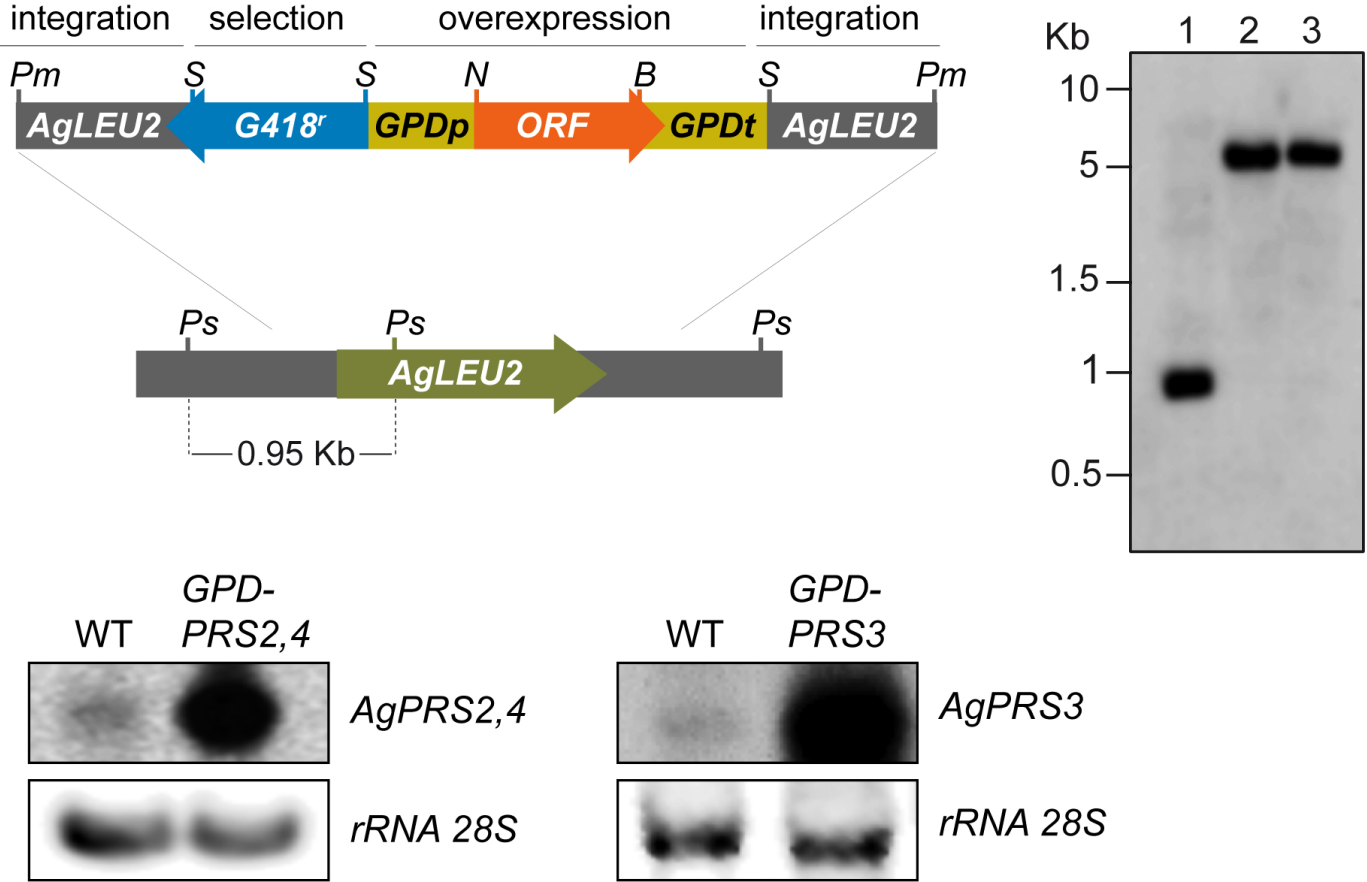
**Overexpression of *A. gossypii PRS2,4 *and *PRS3 *genes**. Top-left, scheme of the modular cassette used for *PRS2,4 *and *PRS3 *overexpression. The integration, selection and overexpression modules are indicated. Top-right, Southern blot analysis to confirm correct integration of the overexpression cassettes into *AgLEU2 *locus. Genomic DNA of the wild-type (lane 1), *GPD-PRS2,4 *(lane 2) and *GPD-PRS3 *(lane 3) strains was digested with *Pst*I and a genomic *Pst*I fragment was used as a radioactive probe. Pm, *Pme*I; S, *Sal*I; N, *Nde*I; B, *Bam*HI; Ps, *Pst*I. Bottom, northern blot analysis of *A. gossypii *total RNA (25 μg) obtained from cultures of the wild-type, *GPD-PRS2,4 *and *GPD-PRS3 *strains grown in MA2 rich medium. rRNA 28S was used as a loading control.

We therefore decided to strongly enhance the expression of *PRS2,4 *and *PRS3 *and analyze the effect of their overexpression on riboflavin production. Accordingly, we designed a stable overexpression cassette based on the promoter and terminator sequences of *AgGPD *(see Materials and Methods for details) (Fig. 6). This cassette allows stable genomic integration into the *AgLEU2 *locus and selection for *G418*^*r*^. *PRS2,4 *and *PRS3 *ORFs were PCR-amplified as *Nde*I-*Bam*HI fragments and were inserted into the overexpression module. Following this, the final constructs were used to transform spores of the *A. gossypii *ATCC 10895 strain and heterokaryotic transformants were selected in medium containing geneticin. After sporulation and clonal selection, homokaryotic *G418*^*r *^transformants were obtained and checked for their leucine auxotrophy. Additionally, integration into the *AgLEU2 *locus of each overexpression cassette was confirmed by Southern-blotting (Fig. 6).

Transcriptional analysis of the *GPD-PRS2,4 *and *GPD-PRS3 *strains by northern-blotting revealed that mRNA levels of both genes were increased by 30-fold (Fig. 6). However, the increase in PRPP synthetase activity did not correlate with the transcriptional levels (see Table 1), suggesting a regulatory mechanism of the enzymatic activity, as described for other PRPP synthetases [16-18]. Nevertheless, when we quantified the riboflavin yield of the strains overexpressing *PRS2,4 *and *PRS3 *we observed a significant improvement in the riboflavin production of both strains (Table 1).

### Effect of enzymatic deregulation of AgPRS2,4 and AgPRS3

PRPP synthetases are subject to feed-back regulation by ADP. Furthermore, the amino acids directly involved in inhibitor binding have been elucidated in several PRPP synthetases [21,29] and it has been reported that specific subtitutions of the leucine 128 and histidine 192 residues of human PRS1 induce allosteric deregulation and enzyme superactivity [9]. In the *A. gossypii *Prs2,4 and Prs3 proteins, those residues are strictly conserved and correspond to leucines 133 and 132; and histidines 196 and 195 in the Prs2,4 and Prs3 proteins respectively.

We therefore designed mutagenic primers (see Additional file 1) to introduce two point mutations in both *PRS2,4 *and *PRS3 *genes. The amino acid substitutions carried out were as follows: the leucines at positions 133 and 132 in Prs2,4 and Prs3, respectively, were substituted by an isoleucine; and the histidines at positions 196 and 195 in Prs2,4 and Prs3 proteins were changed to glutamine. As previously mentioned, both transversions have been reported to cause PRPP synthetase superactivity in humans [9]. After we had amplified the mutant *prs2,4 *and *prs3 *ORFs by mutagenic PCR, we cloned the mutant alleles (*prs2,4-IQ *and *prs3-IQ*) in the overexpression cassette described above and followed the same strategy to integrate them into the *AgLEU2 *locus. Correct genomic integrations in homokaryotic transformants were verified by Southern-blotting (not shown).

**Figure 7:**
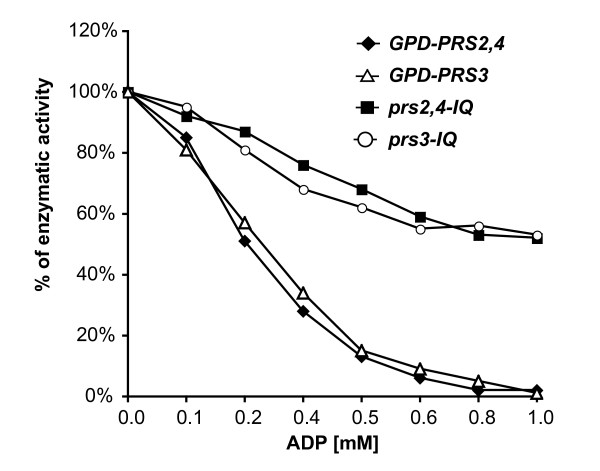
**Effect of overexpression of *A. gossypii PRS *alleles on PRPP synthetase activity and ADP inhibition**. The inhibitory effect of increasing concentrations of ADP was determined using protein extracts from different strains of *A. gossypii*: *GPD-PRS2,4*, *GPD-PRS3*, *prs2,4-IQ *and *prs3-IQ*. Results are means of three independent experiments.

The functionality of the amino acid substitutions were analyzed by PRPP synthetase enzymatic assays in the *prs2,4-IQ *and *prs3-IQ *mutant strains, testing the inhibitory effect of different concentrations of ADP. As shown in figure 6, PRPP synthetase activity was strongly inhibited by ADP in the *GPD-PRS2,4 *and *GPD-PRS3 *strains, while the PRPP synthetase activity of the strains carrying the mutant isoforms prs2,4-IQ and prs3-IQ was highly refractory to the inhibitory effect of ADP. Finally, we analyzed the production of riboflavin in the *prs2,4-IQ *and *prs3-IQ *strains to compare it with that obtained in the wild-type and also in the strains overexpressing the wild-type *PRS2,4 *and *PRS3 *genes. Both strains harboring the deregulated prs2,4-IQ and prs3-IQ attained fairly high production levels, with an 80% greater riboflavin yield than the wild-type strain (Fig. 7). However, riboflavin production did not increase substantially in the *prs2,4-IQ *and *prs3-IQ *strains with respect to the yield obtained after the overexpression of the wild-type alleles, suggesting that other regulatory mechanisms may affect the PRPP intracellular pool and riboflavin production.

## Discussion

Riboflavin is currently employed as an additive in human foodstuffs and animal feeds. Indeed, the industrial production of riboflavin exceeds 3000 tons/year, and the chemical production has now been replaced by biotechnological processes using the natural overproducer *A. gossypii *[28].

Riboflavin biosynthesis relies on the purine pathway, because GTP is the immediate precursor. Accordingly, it seems reasonably that redirecting metabolic flux toward purine biosynthesis might improve riboflavin production. In fact, previous studies described that supplementation with riboflavin precursors can enhance the production of the vitamin in *A. gossypii *cultures [3,31]. PRPP is an essential metabolite in the *de novo *and *salvage *purine pathways and it has been reported that a considerable fraction of the intracellular pool of PRPP is consumed by the purine pathways [7]. We therefore explored the effect of increasing the activity of PRPP synthetase on riboflavin production in *A. gossypii*.

*AGL080C *gene is a syntenic ortholog of *PRS3 *from *S. cerevisiae *and their gene products share 88% of the amino acids. On the other hand, *AGR371C *shows a high degree of similarity with both *PRS2 *and *PRS4 *from *S. cerevisiae *and displays 80% of identity at protein level. Furthermore, *AGR371C *is flanked by the *UBP9 *and *UBC6 *genes, thus exhibiting a high level of synteny with regard to the genomic locations of *PRS2 *and *PRS4 *in *S. cerevisiae*. This redundancy between *PRS *genes and their chromosomal synteny supports the idea that a genome duplication event of ancestral yeast genome would have occurred to originate the *S. cerevisiae *genome [32].

The homology between *PRS *genes from *A. gossypii *and *S. cerevisiae *together with our results concerning the *AgPRS2,4 *and *AgPRS3 *disruptions indicate that two PRPP synthetase interaction complexes may also exist in *A. gossypii*. We found three possible phenotypes when *AgPRS2,4 *and *AgPRS3 *disruptions were combined: synthetic lethality for the double mutant, a growth alteration and an impairment in PRPP synthetase activity for the *Δprs3 *strain, and a slight reduction in total Prs activity that did not elicit phenotypic alterations in the *Δprs2,4 *mutant. Thus, similarly to what occurs in yeast it could be speculated that in *A. gossypii *Prs2,4 and Prs3 function as two different PRPP synthetase units that interact with Prs5 and Prs1, respectively, to yield physiologically specialized enzymatic complexes. The different phenotypes obtained with *AgPRS2,4 *and *AgPRS3 *disruptions can be explained in terms of a different contribution of each isoform to the specific PRPP synthetase functional unit.

Another important issue concerning *AgPRS3 *disruption is that the *Δprs3 *strain displayed an abnormal highly branched growth pattern. Previous work has described that Prs gene products are able to interact with a number of proteins and to affect different biological processes and, in particular, cell integrity signaling through Rlm1. As a consequence, perturbations in the expression of *PRS *genes may result in many unexpected cellular events, such as an abnormal increased chitin content and other cell wall alterations [33-35]. It has also been described that the mechanisms controlling cell polarity, and therefore hyphal morphogenesis, are fairly homologous between *S. cerevisiae *and *A. gossypii *[36]. It therefore seems reasonable to posit that alterations in cell polarity and cell wall morphogenesis might affect the branching pattern of the *A. gossypii *hyphae.

As mentioned above, the altered branching pattern of the *Δprs3 *strain could account for the condensed phenotype of the colonies that finally resulted in a slightly higher biomass. In contrast, the opposite effect has been reported when either *AgPRS2,4 *or *agprs2,4-IQ *alleles are overexpressed in *Arabidopsis thaliana *and *Nicotiana tabacum *[37], demonstrating that variations in the PRPP synthetase activity may have pleiotropic effects. Perhaps the occurrence and interaction of fairly heterologous isoforms of PRPP synthetases from *A. gossypii *together with endogenous isoforms of plant origin or other proteins in the plant cell could provide an explanation for the effect on biomass accumulation.

Our results demonstrate that the PRPP synthetases from *A. gossypii *are inhibited by ADP. The abolishment of ADP inhibition in *A. gossypii *and overexpression of *PRS *genes result in: i) an increase in PRPP synthetase activity and ii) improved riboflavin production, which was the main objective of the present work. However, unlike the overexpression of *PRS3 *alleles, the increase in the levels of either *PRS2,4 *or *prs2,4-IQ *mRNA was not correlated with a marked rise in PRPP synthetase activity, confirming that *PRS2,4 *and *PRS3 *contribute unequally to total Prs activity. However, an improvement of 80% in the production of riboflavin was obtained in the *PRS*-engineered strains, which is clearly of significance as regards biotechnological endeavors. Nonetheless, we failed to find a significant enhancement in the production of the vitamin when the *prs-IQ *alleles were expressed. This may be explained in terms of the existence of a strict transcriptional and metabolic regulation of the purine pathway downstream from the synthesis of PRPP, reported previously in *A. gossypii *and *S. cerevisiae *[3,4,38-42]. In addition, it is also to be expected that the intracellular concentration of ADP in the *PRS*-overexpressing strains would be insufficient to cause inhibition and this would therefore account for the analogous behaviour of the overexpressing mutant strains as compared with the wild-type overexpressing strains.

## Conclusion

In this study we have demonstrated that partially increasing enzymatic PRPP synthetase activity results directly in enhanced riboflavin overproduction in *A. gossypii*. We have engineered *A. gossypii *strains that show a significant improvement in vitamin production. In terms of industrial production, a 80% increase in the fermentation process represents an extremely relevant advance. Further manipulations might be performed to entirely deregulate the purine pathway and to increase GTP availability and hence riboflavin production.

We also show that different mutimeric interacting PRPP synthetase complexes may exist in *A. gossypii*, as described for the *S. cerevisiae *enzymes. Additionally, we prove that modifying PRPP synthetase activity in *A. gossypii *affects its growth pattern, suggesting that homeostasis of the PRPP intracellular pool must be important for the cell integrity and mycelial growth of *A. gossypii*.

## Authors' contributions

JLR conceived the pivotal idea of the study. JLR and MAS co-designed the experiments and supervised the work. AJ performed the experiments and wrote the manuscript. All authors have read and approved the final version of the manuscript.

## Supplementary Material

Additional file 1 List of primers used in this study Click here for file

Additional file 2 Alignments of PRS proteins. Underlined in red are the residues involved in ribose-5-phosphate binding; underlined in blue are the residues involved in pyrophosphate binding. Red circles indicate residues that have been described to participate in catalysis in the *B. subtilis*, *E. coli *and human PRPP synthetases. Click here for file
