# Supplementary material for: Phosphoribosyl pyrophosphate synthetase activity affects growth and riboflavin production in Ashbya gossypii
Source: BMC Biotechnol. 2008 Sep 9;8:67. doi: 10.1186/1472-6750-8-67 (PMC2551608; doi:10.1186/1472-6750-8-67)
Supplement: Additional file 1 — List of primers used in this study [file 1472-6750-8-67-S1.pdf]

Additional file 1. Primers used in the study.

Purpose	*Primer name*	*Sequence*[a]
Cloning	1295-A	5’-GCA AGG ACG GGG ACA AGT-3’ (F)
	1310-A	5’-TTC GCC AAT AAT CAA CTA-3’ (R)
	1311-A	5’-CCT GTC TGG ATG ATG TAG-3’ (F)
	5'1878	5’-GGA TTG TTA CCG GGG AAG GG-3’ (F)
	3'1878	5’-CGT GGT GAA GTA TAT CAA GG-3’ (R)
Expression	AgPRS2,4-Nde	5’-GGA ATT CCA TAT GTC GTC CAA TAG CAT A-3’ (F)
	AgPRS2,4-Bam	5’-GGG ATC CTA CAT GAC AGC GTT ATT G-3’ (R)
	AgPRS3-Nde	5’-GGC ATA TGG CTA CTA ATG CAA TCA AG-3’ (F)
	AgPRS3-Bam	5’-CGG ATC CAA AAT CAT AGT GGG TTG-3’ (R)
	AgLEU2-Sal	5’-CCC CGT CGA CCA ACT GCC CCC AGC AGT ACC-3’ (R)
	AgLEU2-Pme	5’-CCC CGT TTA AAC ACG CAT TCT TGC CTA GGC-3’ (F)
	AgSPL1-Pme	5’-GGG GGT TTA AAC GGC TCC GCA GAC CAT CGC-3’ (R)
	AgSPL1-Sal	5’-GGG GGT CGA CGT TGG CGT TAA ATA GGC AGT-3’ (F)
	BKSP-a	5’-GAG GTT TAA ACC CAG CTT TTG TTC CCT TTA-3’ (F)
	BKSP-b	5’-GGG TTT AAA CCT CCA ATT CGC CCT ATA GTG-3’ (R)
	GPDt-Bam	5’-GGG GGA TCC GCC CGC TAA GCC GGG GGC TTG-3’ (R)
	GPDt-Xho	5’-CCC CTC GAG TTT CCG GTA TGG CGA GCT GTG-3’ (F)
	GPDp-Sal	5’-GGG GTC GAC GGT GTC TGG GTG CAC GAC AC-3’ (F)
	GPDp-Nde	5’-CCC ATA TGT GTG GAC TGA ATT AAT TCA AAC-3’ (R)
Mutations	PRS2,4L133Ia	5’-ATC ACG ATG GAT ATC CAC GCG TCT CAA-3’ (F)
	PRS2,4L133Ib	5’-TTG AGA CGC GTG GAT ATC CAT CGT GAT-3’ (R)
	PRS2,4H196Qa	5’-TTC GCG TTG ATC CAG AAA GAA CGG CAG-3’ (F)
	PRS2,4H196Qb	5’-CTG CCG TTC TTT CTG GAT CAA CGC GAA-3’ (R)
	PRS3L132I	5’-CCA TGG ACA TAC ACG CTT CG-3’ (F)
	PRS3H195Q	5’-GCG CTG ATT CAG AAG GAA CG-3’ (F)

[a]Sequences of forward (F) and reverse (R) primers.
