# Supplementary material for: Phosphoribosyl pyrophosphate synthetase activity affects growth and riboflavin production in Ashbya gossypii
Source: BMC Biotechnol. 2008 Sep 9;8:67. doi: 10.1186/1472-6750-8-67 (PMC2551608; doi:10.1186/1472-6750-8-67)

alignment of Prs proteins

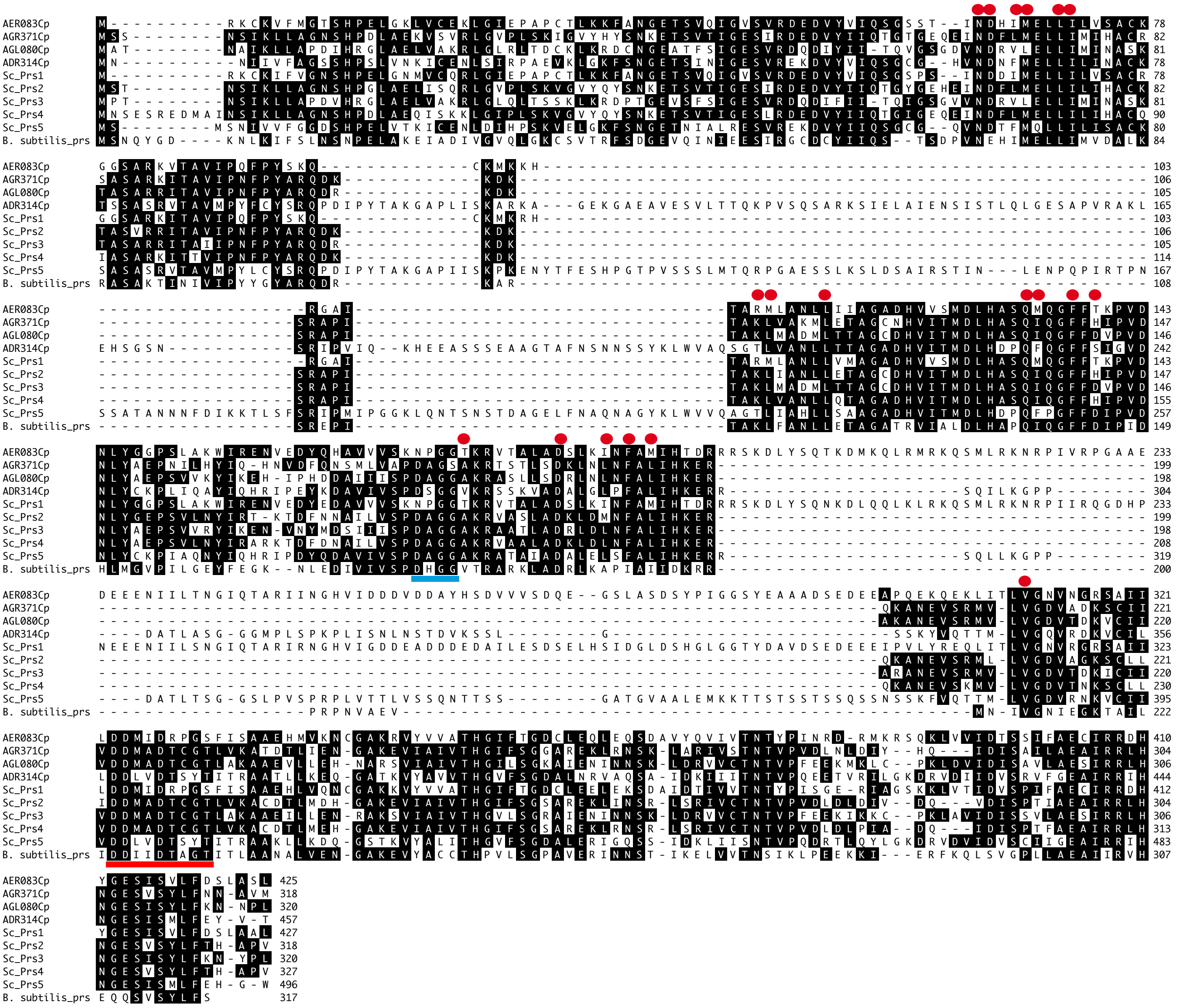

alignment of Prs3 proteins

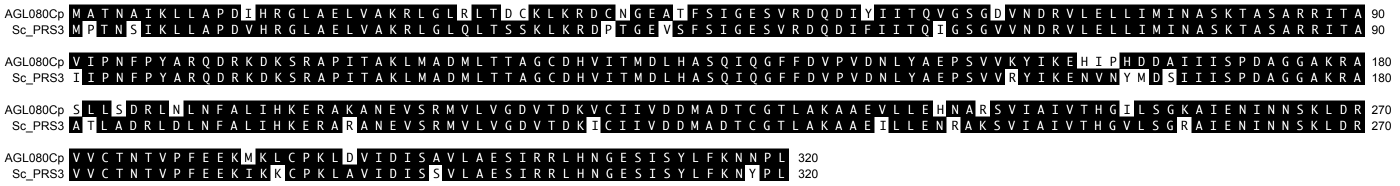

alignment of Prs2 and Prs4 proteins

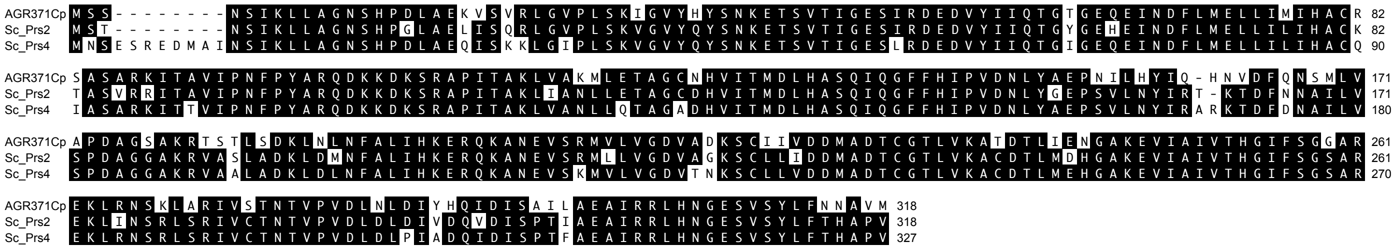

Supplement: Additional file 2 — Alignments of PRS proteins. Underlined in red are the residues involved in ribose-5-phosphate binding; underlined in blue are the residues involved in pyrophosphate binding. Red circles indicate residues that have been described to participate in catalysis in the B. subtilis, E. coli and human PRPP synthetases. [file 1472-6750-8-67-S2.pdf]
